# Mannan-binding lectin regulates dendritic cell maturation and cytokine production induced by lipopolysaccharide

**DOI:** 10.1186/1471-2172-12-1

**Published:** 2011-01-01

**Authors:** Mingyong Wang, Yani Zhang, Yue Chen, Liyun Zhang, Xiao Lu, Zhengliang Chen

**Affiliations:** 1 Department of Immunology, Southern Medical University, Guangzhou, 510515, PR China; 2 Department of Medical Tests, Xinxiang Medical University, Xinxiang, 453003, PR China

## Abstract

**Background:**

Mannan-binding lectin (MBL) is a pattern-recognition molecule present in serum, which is involved in the innate immune defense by activating complement and promoting opsonophagocytosis. Dendritic cells (DCs) are professional antigen presenting cells (APCs) that are crucial for the initiation of adaptive immunity. Lipopolysaccharide (LPS) has been shown to be a strong activator of the inflammatory response and immune regulation. We first examined whether MBL modulated LPS-induced cellular responses, then investigated possible mechanisms of its inhibitory effect.

**Results:**

MBL at higher concentrations (10-20 μg/ml) significantly attenuated LPS-induced maturation of monocyte-derived DCs (MDCs) and production of proinflammatory cytokines (e.g., IL-12 and TNF-α), and inhibited their ability to activate allogeneic T lymphocytes. It bound to immature MDCs at physiological calcium concentrations, and was optimal at supraphysiological calcium concentrations. MBL also bound directly to immature MDCs and attenuated the binding of LPS to the cell surfaces, resulting in decreased LPS-induced nuclear factor-κB (NF-κB) activity in these cells.

**Conclusion:**

All these data suggest that MBL could affect the functions of DCs by modifying LPS-induced cellular responses. This study supports an important role for MBL in the regulation of adaptive immune responses and inflammatory responses.

## Background

Mannan-binding lectin (MBL), a member of the collectin family in the C-type lectin superfamily, is a multimeric protein containing collagen-like sequences. It is synthesized and secreted into the blood by hepatocytes. It has the overall 'bundle-of-tulips' structure first described for C1q [1]. The MBL polypeptide comprises four domains: a cysteine-rich N-terminal domain, a collagen-like region (CLR) that contains Gly-X-Y repeats (where X is any amino acid and Y is often hydroxyproline or hydroxylysine), a neck region and a C-terminal carbohydrate-recognition domain (CRD) [2-7]. The MBL polymer is composed of as many as six homogenous subunits [8], but only the more highly polymeric forms have biological activity and can fix complement.

Dendritic cells (DCs) are highly efficient antigen-presenting cells that play a central role in orchestrating adaptive immune responses to pathogens [9]. In their immature form (imDCs), they can capture antigen very efficiently by macropinocytosis, endocytosis [10], and phagocytosis [11] through different cell surface molecules, e.g. mannose receptor, Toll-like receptors (TLRs) or scavenger receptors. To elicit an immune response, DCs must undergo a maturation process, which is initiated by inflammatory signals and is completed after contact with T cells. Maturation enables DCs to migrate from peripheral tissues to lymphoid organs and to acquire a very potent antigen-presenting capacity. Mature DCs are also the initial and most prominent source of the cytokines that govern the development of Th1 responses (e.g., IL-12). DC maturation is known to be induced by several stimuli, including bacterial components [e.g., lipopolysaccharide (LPS)], heat shock proteins, viral products (e.g., dsRNA) and endogenous factors such as inflammatory cytokines [12].

It has been reported that other members of the soluble defensive collagen family such as surfactant protein A (SP-A), surfactant protein D (SP-D) and complement C1q, have roles in immune regulation by interacting with various cells. For instance, SP-A inhibits the differentiation and maturation of DCs [13] and modulates cellular responses induced by LPS through interacting with CD14 [14]; SP-A suppresses TNF-α secretion from alveolar macrophages stimulated by LPS [15], and SP-A-deficient mice produce significantly more TNF-α than wild-type mice after intratracheal LPS administration [16]. SP-D enhances bacterial antigen presentation by bone marrow-derived DCs [17]. C1q regulates LPS-induced cytokine production in bone marrow-derived DCs [18] and induces maturation of human DCs [19].

As a key soluble pattern recognition molecule in the innate immune system, MBL has been reported to influence the cytokine network after stimulation by various microorganisms [20-23]. To date, however, little knowledge has been obtained about its role in the regulation of adaptive response. Since DCs play key roles in initiating adaptive responses, it will be useful to know whether and how this member of the collectin family regulates LPS-induced biological functions and the possible mechanisms in DCs.

## Results

### Purification of MBL

**Figure 1:**
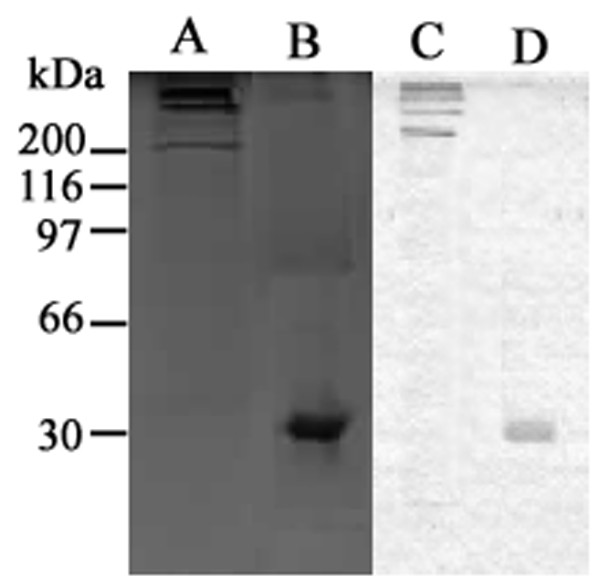
**Analyses of the purified MBL protein by SDS-PAGE and western blotting**. (A) The purified MBL under non-reducing conditions; (B) the purified MBL under reducing conditions; (C) western blot of the purified MBL under non-reducing conditions; (D) western blot of the purified MBL under reducing conditions.

MBL was purified from pooled human plasma samples containing high concentrations (approximately 2500 μg MBL/L on average). SDS-PAGE and western blotting showed that the highly purified MBL was a functional multimer composed of 32KD peptide chains (Figure 1). It was highly bioactive, as demonstrated by a ligand-binding assay and yeast coagulation (data not shown).

### MBL suppresses LPS-induced MDC maturation

**Figure 2:**
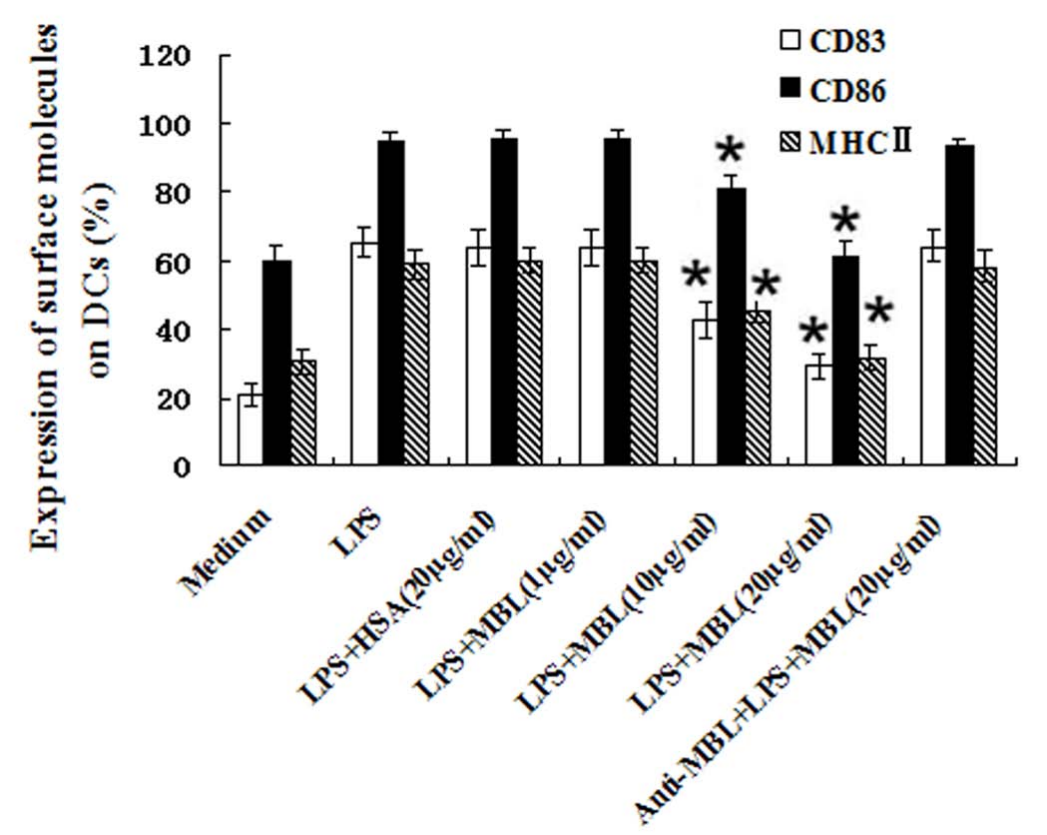
**MBL suppresses LPS-induced MDC maturation**. ImMDCs were stimulated with LPS (100 ng/ml) in the presence of the indicated concentrations of HSA, MBL, or MBL and anti-MBL pAb for 2 days. Cell maturation was assessed by flow cytometry. *P < 0.05 as compared to LPS-stimulated group. Similar results were observed in three independent experiments.

It is well known that DC maturation is induced by LPS. During DC maturation, the expression of receptors required for phagocytosis is down-regulated, whereas MHCII and co-stimulatory molecules such as CD80 and CD86 are up-regulated [24]. To investigate whether MBL influences DC maturation, imMDCs were stimulated with smooth LPS (100 ng/ml) in the presence of increasing concentrations of MBL (0, 1, 10, 20 μg/ml) for 2 days. Cell maturation was assessed by flow cytometry (FCM). As shown in Figure 2, LPS induced elevated expression of CD83, CD86, and MHC II, but these effects were strongly inhibited by MBL at higher concentrations (10-20 μg/ml) compared with the corresponding cells without MBL treatment (*P *< 0.05); HSA (20 μg/ml) and lower MBL concentrations (1 μg/ml) had no such effect. Inclusion of anti-MBL polyclonal antibody (pAb) during the preincubation of the cells with MBL restored the expression of CD83, CD86, and MHC II on DCs (Figure 2), indicating that MBL specifically inhibited the interaction between LPS and imMDCs.

### MBL inhibits TNF-α and IL-12 p40+p70 production from LPS-stimulated MDCs

**Figure 3:**
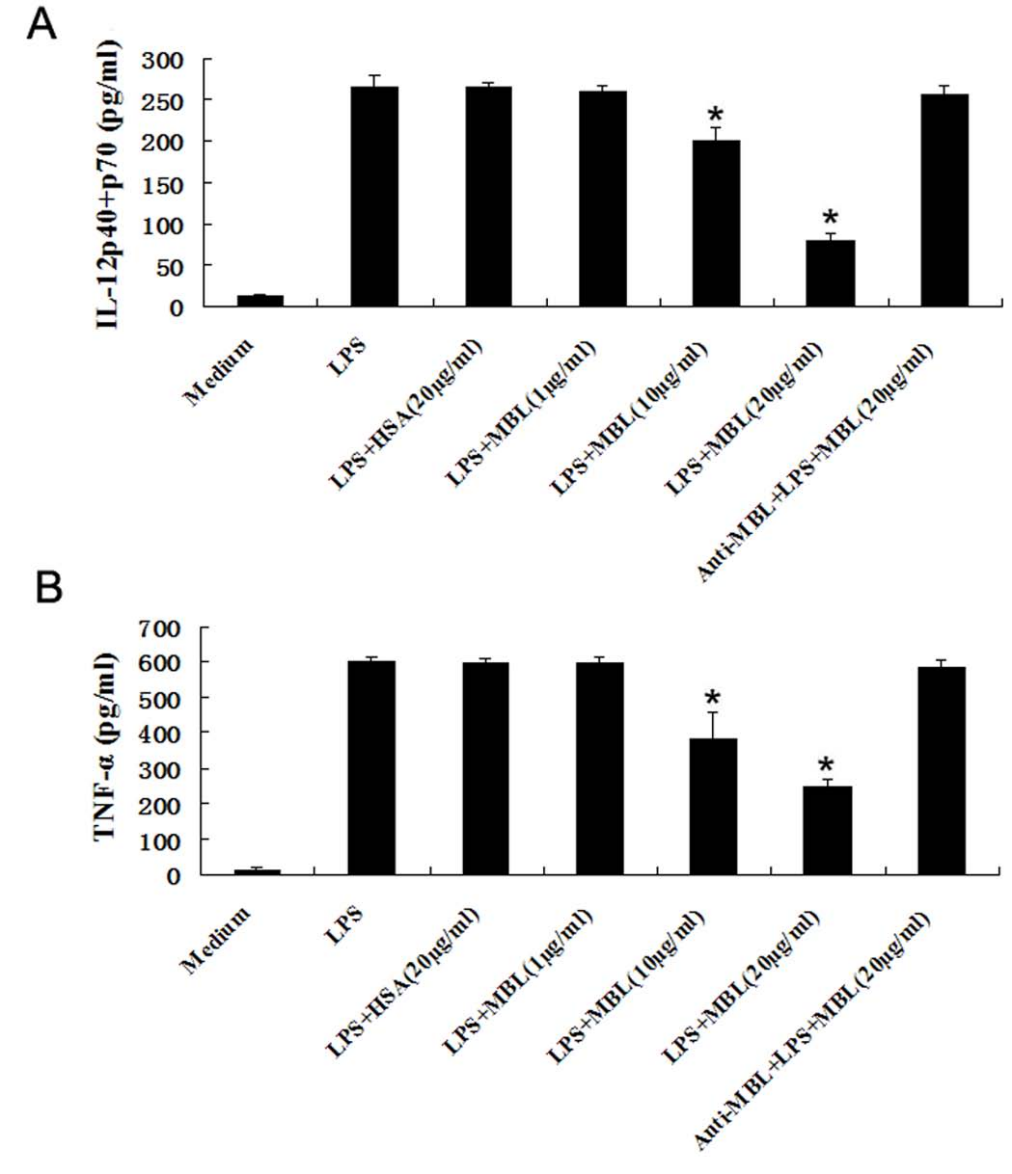
**MBL inhibits LPS-induced cytokine production from imMDCs**. ImMDCs were stimulated with LPS (100 ng/ml) in the presence of the indicated concentrations of HSA, MBL, or MBL and anti-MBL pAb for 24 h. Supernatants were harvested and subjected to ELISA for IL-12 p40+p70(A) and TNF-α (B). *P < 0.05 as compared to LPS-stimulated group. Similar results were observed in three independent experiments.

ImMDCs were stimulated with LPS and exhibited maximal response to secreted proinflammatory cytokines such as IL-12 and TNF-α (data not shown). Cytokines produced by MDCs are known to be important in regulating the adaptive immune response, directing it to either the Th1 or the Th2 pathway. To investigate whether MBL affects cytokine production by MDCs stimulated with LPS, imMDCs were stimulated with LPS (100 ng/ml) in the presence of increasing concentrations of MBL (0, 1, 10, 20 μg/ml) for 24 h, and the supernatants were analyzed by ELISA. As shown in Figure 3, the inductions of TNF-α and IL-12 p40+p70 by LPS were strongly inhibited by MBL at higher concentrations (10-20 μg/ml), compared with the corresponding imMDCs without MBL treatment (*P *< 0.05), but HSA (20 μg/ml) and lower MBL concentrations (1 μg/ml) had no such effect. Inclusion of Anti-MBL pAb during the preincubation of the cells with MBL restored the secretion of TNF-α and IL-12 p40+p70 (Figure 3), indicating that the inhibitory effect of MBL is specific.

### MBL attenuates allogeneic T cell proliferation induced by LPS-stimulated MDCs

**Figure 4:**
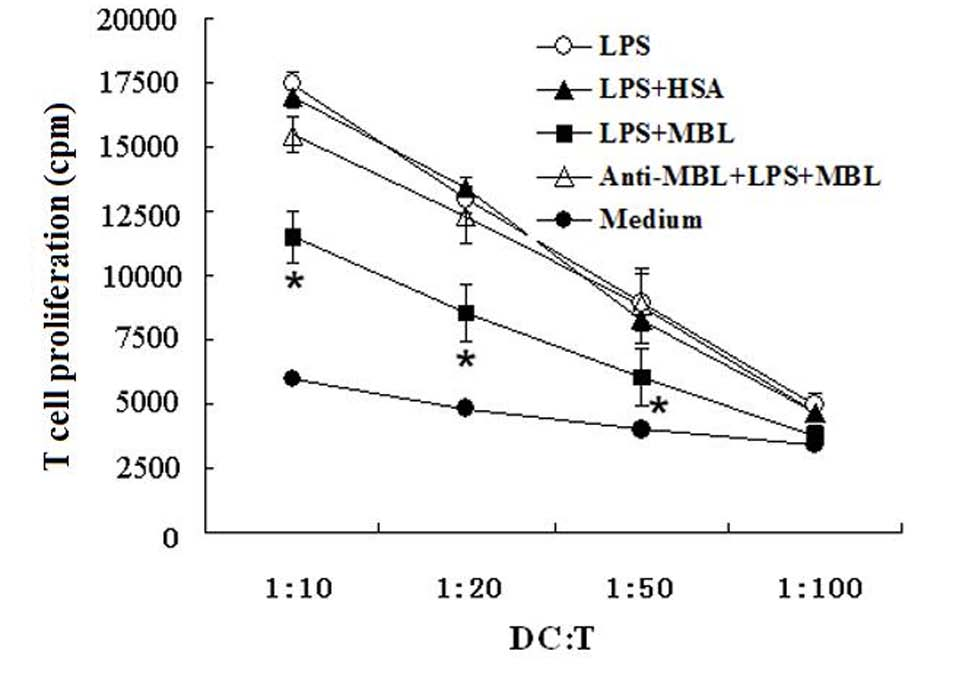
**MBL attenuates allogeneic T cell proliferation induced by LPS-primed MDCs**. T cells obtained from PBMC (2 × 10^5 ^cells/well) were co-cultured with either imMDCs, MBL-preincubated and LPS-primed MDCs, HSA-preincubated and LPS-primed MDCs, or LPS-primed MDCs at the indicated DC:T cell ratios. Anti-MBL pAb was used to demonstrate the specificity of the MBL-induced response. Proliferation of T cells was determined after 4 days of culture by ^3^H-thymidine uptake. Data given as mean ± S.D. of triplicate samples are representative of four independent experiments. *P < 0.05 as compared to LPS-stimulated group.

As mature DCs are known to be the most potent T cell activators, we next tested whether MBL affects the proliferative response of allogeneic T cells induced by LPS-stimulated MDCs. As demonstrated in Figure 4, T lymphocytes induced by LPS-stimulated MDCs co-cultured with MBL (15 μg/ml) showed significantly lower proliferation than the corresponding MDCs without MBL treatment (*P *< 0.05); HSA (15 μg/ml) had no effect. In control samples, MDCs incubated in medium only or primed with LPS were used for the mixed lymphocyte reaction. Inclusion of anti-MBL pAb during the preincubation of the cells with MBL restored allogeneic T cell proliferation (Figure 4), further indicating the specific inhibitory effect of MBL.

### MBL attenuates the binding of LPS to imMDCs

**Figure 5:**
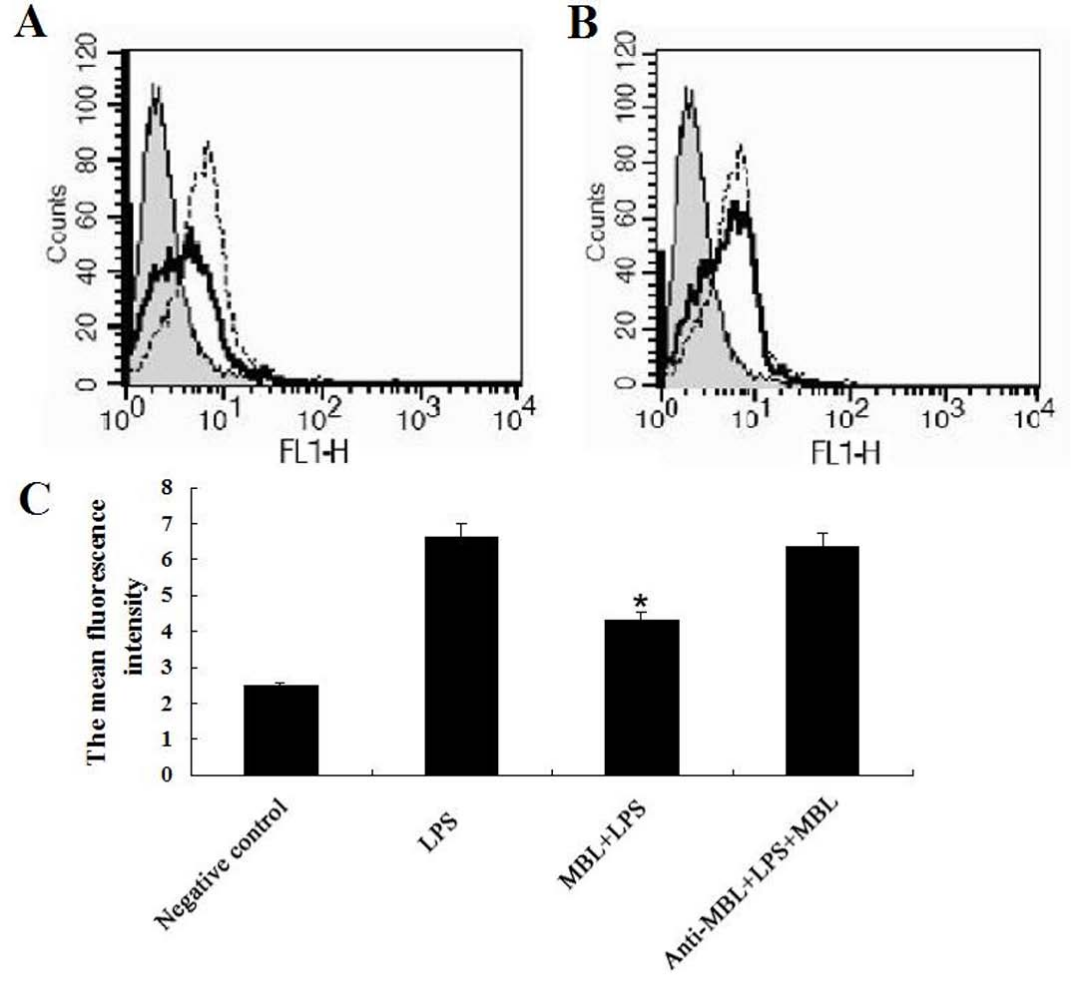
**MBL attenuates the binding of smooth LPS to imMDCs**. ImMDCs were preincubated with (black solid line) or without (dotted line) MBL (10 μg/ml) for 30 min (A), or with anti-MBL pAb (30 μg/ml) and MBL (black solid line) for 30 min (B), and further incubated at 4°C for 30 min with Alexa488-labeled smooth LPS (*E. coli *O111: B4). The binding of LPS on the cell surface was determined by FACScan. The histograms shown are representatives from three experiments. Shaded curves, the negative control without labeled LPS. (C) Mean fluorescence intensity, data given as mean ± S.D. from three independent experiments. *P < 0.05 as compared to LPS group.

We next examined whether MBL altered LPS binding to imMDCs. When the cells were incubated at 4°C with labeled smooth LPS (Figure 5A), significant LPS binding was observed on the cell surface (Figure 5A, dotted line). After the cells were preincubated with MBL (10 μg/ml), this cell-surface binding of the labeled smooth LPS was significantly attenuated (Figure 5A, black solid line). Inclusion of anti-MBL pAb during the preincubation of the cells with MBL restored the binding of smooth LPS to the cell surface (Figure 5B), indicating that MBL specifically inhibits the interaction between smooth LPS and the imMDCs.

We also checked whether MBL itself can bind to the LPS used in present experiments. The results showed that MBL bound to rough LPS from *E. coli *O111: B4 mutant (J5) but not to smooth LPS from *E. coli *O111: B4 (Additional file 1, Fig. S1), suggesting that the MBL-imMDCs interaction rather than an MBL-LPS interaction affects the LPS-induced cellular responses.

### Ca^2+^-dependent binding of MBL to imMDCs

**Figure 6:**
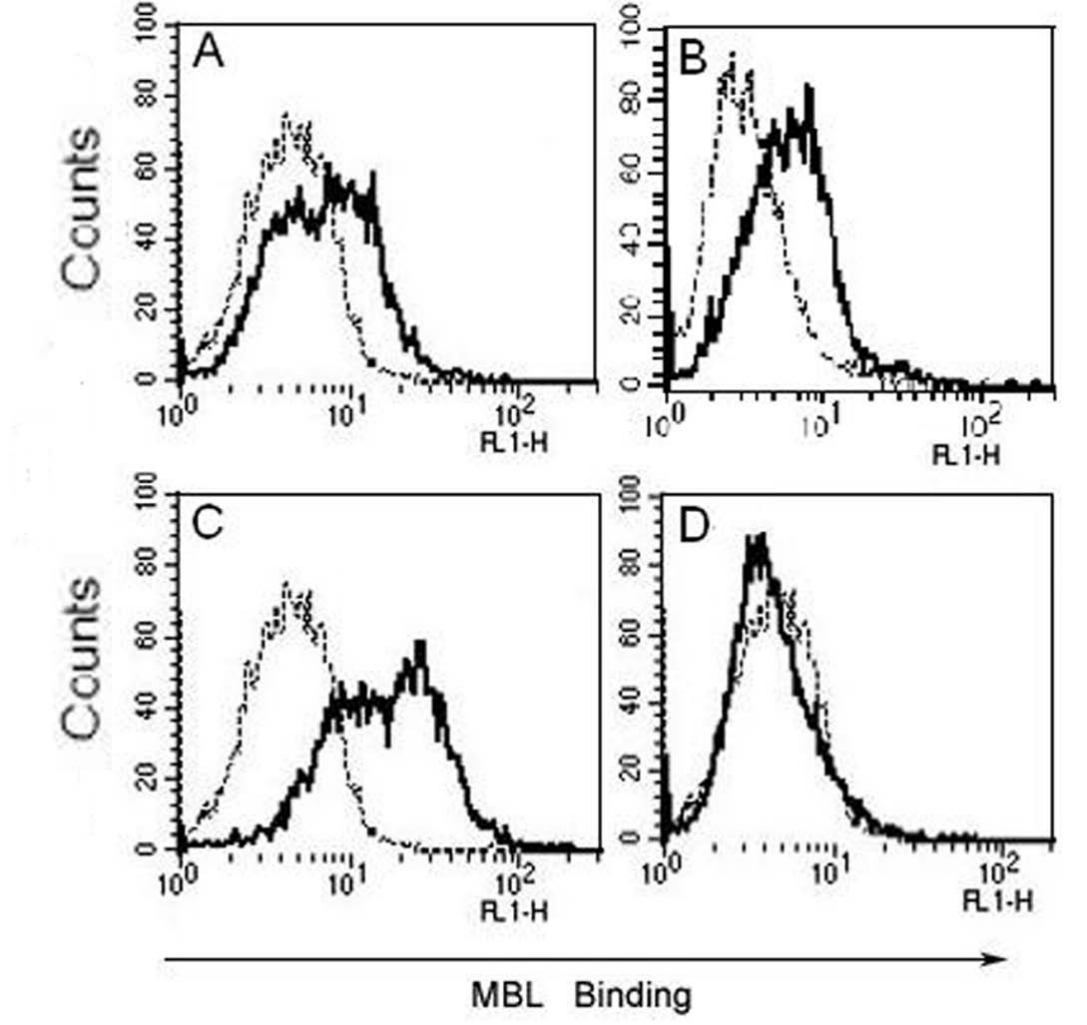
**Ca^2+^-dependent binding of MBL to imMDCs**. (A) Tris-buffered saline with 1.5 mM CaCl_2_; (B) Tris-buffered saline with 5 mM CaCl_2_; (C) Tris-buffered saline with 10 mM CaCl_2_; (D) Tris-buffered saline with 5 mM EDTA. Each solution of imMDCs was added with the biotinylated MBL (10 μg/ml) for 10 min incubation before addition of ExtrAvidin-FITC. After incubation for 30 min on ice, binding of MBL to the cells was analyzed by FACScan, as shown in the representative histograms (A-D). Black lines, biotinylated MBL binding; dotted lines, negative controls (the cells only). These data are representative of four independent experiments.

When imMDCs were incubated with biotinylated MBL at physiological calcium concentrations (buffer A), there was definite but slight MBL binding to the cells, as demonstrated by FCM (Figure 6A). At higher Ca^2+ ^concentrations the binding was markedly increased (Figure 6B,C); also, the binding was markedly reduced in the absence of Ca^2+ ^(Figure 6D). These results showed that MBL interacted with imMDCs in a Ca^2+^-dependent manner.

### MBL inhibits NF-κB DNA-binding and translocation in imMDCs

**Figure 7:**
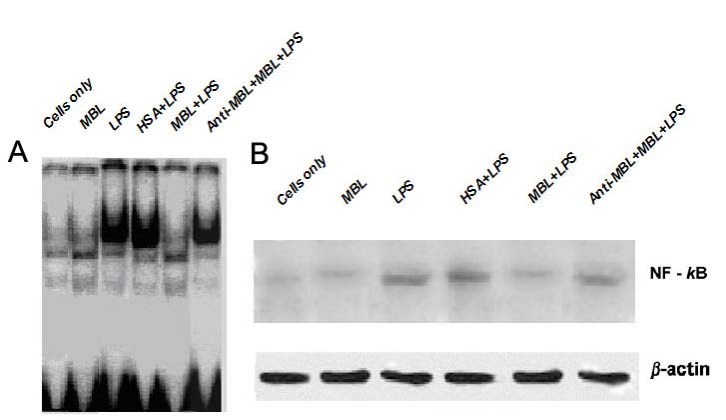
**MBL decreases LPS-stimulated NF-κB binding activity and translocation in imMDCs**. (A) LPS-induced DNA-binding activity of NF-κB is inhibited by MBL. ImMDCs (5 × 10^5 ^cells/sample) were stimulated with LPS (100 ng/ml) in the presence of 15 μg/ml of HSA, anti-MBL pAb and MBL, or MBL for 1 h, then harvested to prepare nuclear extracts. The nuclear extracts were mixed with radiolabeled NF-κB oligonucleotide probe and analyzed with EMSA. (B) MBL inhibits NF-κB translocation in imMDCs. ImMDCs (5 × 10^5 ^cells/sample) were stimulated with LPS (100 ng/ml) in the presence of 15 μg/ml HSA, anti-MBL pAb and MBL, or MBL for 1 h, then the cells were harvested to prepare nuclear extracts. The proteins in the nuclei-free supernatants were separated by 10% SDS-PAGE, followed by transfer to a nitrocellulose membrane. After blocking, the membrane was incubated with the NF-κB-specific mouse anti-human mAb p65, followed by HRP-conjugated secondary antibody. ECL was used to visualize the protein bands. As an internal control, actin was used.

The next question is whether MBL modulates LPS-induced signaling as a consequence of its interaction with surface molecules on imMDCs. Therefore, we investigated the effects of MBL on LPS-induced NF-κB activation in imMDCs, using two methods: electrophoretic mobility shift assay (EMSA) and western blotting. For EMSA, when nuclear extracts from LPS-stimulated imMDCs were incubated with p40 NF-κB probe, NF-κB binding was enhanced within 1 h after LPS-stimulation, and MBL (15 μg/ml) reduced the DNA binding activity; DNA binding was restored by including anti-MBL pAb (Figure 7A). This binding of the complex was specific because it could be competed away with a 100-fold molar excess of unlabeled consensus NF-κB oligonucleotide (data not shown). For western blotting, analysis of corresponding nuclear fractions using anti-p65 Ab confirmed that LPS significantly increased NF-κB translocation from the cytoplasm to the nucleus in imMDCs, and treatment with 15 μg/ml MBL strongly inhibited this effect (Figure 7B). Inclusion of anti-MBL pAb restored NF-κB translocation from the cytoplasm to the nucleus (Figure 7B), further indicating the specific inhibitory effect of MBL on the interaction between smooth LPS and the imMDCs.

## Discussion

This study demonstrates a new biological function of MBL in regulating LPS-induced MDCs maturation and cytokine production. MBL bound to imMDCs at physiological calcium concentrations, but binding was optimal at supraphysiological concentrations. Furthermore, direct binding of MBL to imMDCs attenuated the binding of LPS to the cell surface and decreased LPS-induced NF-κB activity, suggesting that it affected MDC functions by modifying the LPS-induced cellular responses.

DCs are professional APCs that are crucial in the initiation of adaptive immunity [12]. They recognize pathogens through pattern recognition receptors (PRRs) that are triggered by pathogen-associated molecular patterns, leading to DC maturation. Maturation is a key process in DC biology and determines whether an immune response or tolerance will be initiated upon interaction with a given antigen. It is well accepted that imDCs induce T cell tolerance by inducing regulatory T cells [25]. To determine whether MBL affects LPS-induced MDC maturation, imMDCs were cultivated with MBL at a range of final concentrations from 0 to 20 μg/ml. The results showed that cells treated with high MBL concentrations (10-20 μg/ml) expressed significantly less CD83, CD86, and MHC II than the corresponding MDCs stimulated with LPS only. In addition, the proliferation of allogeneic T lymphocytes induced by LPS-treated MDCs was significantly inhibited when they were co-cultured with 15 μg/ml MBL, suggesting that MBL could regulate adaptive immune systems by modulating DC maturation.

Recently Macdonald *et al*. [26] reported that rhMBL did not directly influence DC differentiation or maturation. Mature DCs, prepared in the presence of rhMBL and subsequently co-cultured with allogeneic mononuclear cells, markedly promoted the production of IL-1, IL-6 and TNF-α *in vitro*. This influence required the presence of rhMBL during DC maturation and was critically dependent on the presence of monocytes. However, the data presented in this study demonstrate that MBL suppresses DC maturation and cytokine secretion in the presence of LPS *in vitro*, suggesting that MBL may act differently under different (physiological or pathological) conditions and shows much versatility in regulating cellular immunity in addition to its established role as an opsonin.

It has been recognized that the nature of the innate immune response significantly influences the nature of the subsequent adaptive immune response. Both soluble and membrane-bound PRRs of the innate immune system assess the level of danger of a particular intrusion and initiate a program of protection for the host [27]. Phagocytic cells (e.g., monocytes, macrophages and DCs) mediate some of these changes, when activated by pathogens, which often initiate the synthesis of proinflammatory cytokines [28]. The cytokine environment then influences the subsequent specific immune responses. This study provides strong evidence that MBL at higher concentrations (10-20 μg/ml), but not at lower concentrations (1 μg/ml), significantly inhibits LPS-induced TNF-α and IL-12 p40+p70 production, indicating that could affect the adaptive immune response by modulating the cytokine network.

It is known that mouse MBL-A interacts with the rough but not the smooth LPS [29]. Estabrook *et al*. [30] and Sprong *et al*. [31] also proved that human MBL did not interact with LPS. In the present study, smooth LPS was used to avoid interference from any LPS-MBL interaction. We also demonstrated that MBL itself did not bind to the smooth LPS used in this study. This evidence strongly supports the view that the MBL-imMDC interaction rather than the MBL-LPS interaction affects LPS-induced cellular responses.

The functional MBL receptor expressed by many cell types is still controversial. As a major soluble PRR in the innate immune system, MBL has long been known to recognize pathogens or autologous apoptotic cells via its CRD and to interact with autologous cells via its CLR. It was believed that the CRD does not interact with normal autologous cells, but recently Downing *et al*. [32] reported calcium-dependent MBL binding to autogeneic B lymphocytes, monocytes and imMDCs via its C-type lectin-binding site, suggesting a new role of MBL in the immune system. In this study, there was clear evidence to demonstrate that MBL binds imMDCs at physiological calcium concentrations, though the binding was optimal at supraphysiological concentrations. SP-A directly interacts with TLR4 and MD-2 and regulates inflammatory cellular responses [33], and MBL and lung collectins interact with TLR4 and MD-2 by different mechanisms [34]. As LPS/TLR- signaling pathways are crucial for controlling the early inflammatory response and TLR4 is critical for LPS recognition and signaling [35], we presumed that TLR4 might be directly involved in MBL binding to the cell surface. It is also well known that LPS recognition is a complex process involving cooperation of several molecules such as LBP, CD14 and MD2, which enhance TLR4 signaling. Rat MBL might interact with CD14 [29]. Therefore, other cell surface molecules might play a role through binding to MBL. However, the molecular mechanisms by which MBL interacts with cells need to be investigated further.

LPS-activated cells sometimes induce microcirculatory dysfunction as well as inflammatory changes, which cause injury to various tissues, circulatory failure, and occasionally death [36-38]. To prevent excessive and prolonged responses by host innate immune cells to LPS, the host may acquire a down-regulating system, LPS tolerance, which ensures safe responses to LPS and/or unresponsiveness to a second stimulation [39]. The fact that MBL at higher concentrations inhibits LPS-induced DC maturation and proinflammatory cytokine secretion suggests that it could provide such down-regulation in an inflammatory situation. In addition, the MBL preparation used in this study was determined without endotoxin by the Limulus amebocyte lysate assay, ruling out LPS tolerance induced by endotoxin contamination in the MBL preparations.

The serum concentration of MBL normally ranges from 0.01 to 10 μg/ml [40-42], but as an acute-phase protein, this can increase from 1.5- to 3-fold in patients during an acute phase response [43]. The serum levels of MBL in patients with rheumatic heart disease increased significantly up to 14 μg/ml [44]. MBL is also present in such extravascular milieux as synovial fluid, amniotic fluid, lung and vaginal lavages [45-49], and behaves as an acute-phase reactant in individuals with 'sufficient' (wild-type) MBL genotypes [50]. Therefore, we presume that MBL binding to autologous cells is most likely to take place at inflammatory loci, where local elevation of the extravascular MBL concentration and a concomitant influx of immune system cells are to be expected. The data presented in this paper showed that LPS-induced MDC functions were significantly modulated by MBL at higher concentrations (10-20 μg/ml) but not at lower concentrations (1 μg/ml). Therefore, by analogy, we presume that further investigations could provide evidence that, in the acute phase, high-concentration MBL is secreted to affect cytokine-mediated immune regulation in the serum as well as locally in some extravascular tissues.

Therefore, these results suggest that MBL might have anti-inflammatory effects in pathological states. As a common exogenous pathogenic factor, LPS is not only a major mediator in the systemic inflammatory response and sepsis, but also a strong activator of monocytes, macrophages and DCs. During inflammatory shock, LPS stimulates monocytes to produce excessive inflammatory mediators and their cascade reactions, leading to multiple organ failure. Numerous studies have confirmed that the imbalanced cytokines mediating the interaction of natural and acquired immunity contribute to human sepsis and immune dysfunctions. Overproduction of proinflammatory cytokines (e.g. TNF-α, IL-12, IFN-γ and IL-18) results in sustained sepsis, shock, and even death [51,52]. In this study, higher concentrations of MBL inhibited LPS-induced DC maturation and the secretion of such proinflammatory cytokines as TNF-α and IL-12, indicating that MBL might be implicated in the anti-inflammatory effect and immunoregulation, reducing the incidence of shock and prevent endotoxemia-induced death. This study paves the way for a novel treatment of inflammatory shock by modulating excessive activated DCs with MBL.

## Conclusions

In summary, we demonstrated in this study that MBL could attenuate MDCs maturation and cytokine production induced by LPS. MBL also bound directly to imMDCs and attenuated the binding of LPS to the cell surfaces, resulting in decreased LPS-induced nuclear NF-κB activity in these cells. All these data suggest that MBL could affect the functions of DCs by modifying LPS-induced cellular responses. This study supports an important role for MBL in the regulation of adaptive immune responses and inflammatory responses.

## Methods

### Preparation of MBL

MBL was isolated from human plasma according to Tan *et al*. [53], modified as described [54]. Briefly, a pool of freshly frozen human plasma (2.5 liters) (provided by Guangzhou General Hospital of Guangzhou Military Area Command of Chinese PLA, China) was thawed from -80°C and, after extraction and elimination of most of the unrelated proteins, the residual fraction was solubilized and MBL was purified by a process involving three chromatographic steps. The first step was affinity chromatography on a mannan-agarose (Sigma, Poole, UK) column, which selected for functionally active, carbohydrate-binding MBL with about 2000-fold purification. The subsequent steps were anion-exchange chromatography and gel filtration on a Mono-Q HR 5/5 column (Pharmacia Biotech Europe, Orsay, France) and a Superose 6 HR 10/30 column (Pharmacia Biotech, Orsay, France), respectively. The purified MBL was evaluated by SDS-PAGE and western blotting.

The MBL preparation was determined without endotoxin contamination by the Limulus amebocyte lysate assay. Biotinylated MBL was prepared by coupling N-hydroxysuccinimidobiotin to the purified MBL as described [55].

### Preparation of human DCs

DCs were prepared from peripheral blood monocytes of healthy individuals. Briefly, peripheral blood mononuclear cells (PBMCs) were isolated by standard density gradient centrifugation and further separated on multistep Percoll gradients (Pharmacia, Uppsala, Sweden). The cells were suspended in IMDM (Gibco BRL, Gaithersburg, MD, USA) and allowed to adhere to 6-well tissue culture plates (Corning-Costar, MA, USA). After incubation for 40 min at 37°C, non-adherent lymphocytes were removed. Adherent monocytes were harvested and washed twice, then cultivated for 5 days in IMDM containing rHuGM-CSF (115 ng/ml; Peprotech, Inc., Rocky Hill, N.J.) and rHuIL-4 (50 ng/ml; Peprotech, Inc.) and supplemented with 10% (v/v) heat-inactivated fetal calf serum (FCS, Gibco BRL, Grand Island, CA), penicillin (100 U/ml) and streptomycin (100 μg/ml). The cells were kept in culture at a density of 10^6 ^cells/ml in 12-well plates (Corning-Costar, MA, USA) at 37°C in a 5% (v/v) CO_2 _atmosphere. On day 3, additional rHuGM-CSF (115 ng/ml) and rHuIL-4 (50 ng/ml) were added to the cultures. On day 5, imMDCs were harvested by centrifugation at 450 × g for 5 min and washed with IMDM before the experiments.

To study the effect of MBL on maturation and cytokine production, the harvested imMDCs (1 × 10^6 ^cells/ml) were seeded in 12-well plates in complete IMDM, and maintained at 37°C in a 5% (v/v) CO_2 _environment for 2 h after MBL was added to a range of final concentrations (0-20 μg/ml). rHuGM-CSF (115 ng/ml) and rHuIL-4 (50 ng/ml), and smooth LPS (100 ng/ml; from *E. coli *O111: B4, Campbell, CA), were added to the complete medium. Samples were kept at 37°C under a humidified 5% CO_2 _environment for 2 days, and then analyzed. In control groups, human serum albumin (HSA, 20 μg/ml) was used. To demonstrate the specificity of the responses to MBL, anti-MBL pAb (R&D systems, MN, USA) was used.

### Flow cytometric determination of MDC phenotypes

To determine phenotypes, MDCs were washed and then incubated in PBS containing 2% FCS and 0.01% NaN_3, _with the following monoclonal antibodies (mAbs): FITC-conjugated anti-human HLA-DR and FITC-conjugated anti-human CD14 (Becton Dickinson, San Jose, USA), PE-conjugated anti-human CD1a, PE-conjugated anti-human CD86, PE-conjugated anti-human CD80, PE-conjugated anti-human CD83, FITC-conjugated anti-mouse IgG, and control mouse IgG (eBioscience, San Diego, USA). In control samples, the mAbs were substituted with matched isotype control mouse Ig (Becton Dickinson, San Jose, USA). Cells were analyzed in a FACScan equipped with Cell Quest software (Becton Dickinson, Mountain View, USA).

### Cytokine measurements

The supernatants of the various samples were collected and stored at -80°C pending analysis. For negative controls, the cells were cultured in complete medium only. Production of IL-12 p40+p70 and TNF-α were determined using ELISA kits (Bender MedSystems, San Diego, USA), as suggested by the manufacturers.

### Mixed lymphocyte reaction (MLR)

CD3^+ ^T cells were isolated from PBMCs as described [56]. Isolated T cells were transferred to 96-well flat-bottom microplates (Corning-Costar, MA, USA) at a density of 2 × 10^5 ^cells/well. These T cells were co-cultured with LPS-induced DCs preincubated with 15 μg/ml MBL in the indicated DC:T cell ratios, without additional exogenous cytokine. To demonstrate the specificity of responses to MBL, anti-MBL pAb was used. Proliferation of T cells was determined by ^3^H-thymidine (Perkin-Elmer, MA, USA) incorporation. Net counts per minute (cpm) were measured in triplicate cultures.

### Effect of MBL on the binding of LPS to imMDCs

ImMDCs were collected, and 10^6 ^cells/tube were preincubated in the absence or presence of 10 μg/ml MBL at 37°C for 30 min. The cells were then further incubated with 100 ng/ml Alexa488-labeled smooth LPS (*E. coli *O111: B4, the molecular mass is ~10,000 Da, Molecular Probes, MN, USA) at 4°C for 30 min. After washing, the cells were analyzed by FACScan. To demonstrate the specificity of the response to MBL, anti-MBL pAb was added to MBL preparations for 10 min before incubating with the cells.

### Binding of MBL to imMDCs

The ligand binding assay was performed as described [57]. Washed imMDCs (2 × 10^5^) were resuspended in Tris-buffered saline (pH7.4) containing 5 mM CaCl_2_, 1% bovine serum albumin (buffer A). Three kinds of Tris-buffered saline containing different calcium concentrations were alternatively used for binding assays. In the Ca^2+^-free control, 5 mM EDTA was substituted for CaCl_2_. Each cell suspension (0.2 ml) was first incubated for 30 min on ice with either biotinylated MBL or unlabelled MBL. The cells were incubated further for 30 min on ice with ExtrAvidin-FITC (Sigma, Madrid, Spain) at a final dilution of 1 in 100. After washing, the cells were analyzed by FACSCalibur.

The possibility that MBL itself binds to the LPS used in the experiments was also examined using microtiter wells. Smooth LPS (*E. coli *O111: B4), rough LPS [*E. coli *O111: B4 mutant (J5)] or mannan (10 μg/ml, 50 μl/well; Sigma, St. Louis, USA) was coated on to the wells, which were incubated with 10 mM Hepes buffer (pH 7.4) containing 0.15 M NaCl, 5 mM CaCl_2_, and 5% (w/v) BSA (buffer B) to block nonspecific binding. The indicated concentrations of MBL in buffer B were added and incubated at 37°C for 1 h. After washing, the wells were incubated with anti-human MBL mAb (1:5000). Binding was detected with HRP-labeled goat anti-mouse IgG, and the absorbance at 450 nm was measured.

### Analyses of NF-kB by EMSA and western blotting

ImMDCs (5 × 10^5 ^cells/sample) were stimulated with LPS (100 ng/ml) in the presence of 15 μg/ml MBL, HAS, or MBL and anti-MBL pAb for 1 h. The DCs were harvested and nuclear extracts were prepared using the NucBuster protein extraction kit (Novagen, Darmstadt, Germany). Oligonucleotide probes were radiolabeled with [γ-^32^P] ATP by T4 polynucleotide kinase (Takara, Tokyo, Japan). For the binding reaction, 5 μg of nuclear extracts were incubated in 30 μl total reaction buffer containing 10 mM Hepes (pH 7.9), 12.5% glycerol, 70 mM NaCl, 1 mM DTT, 1 mM EDTA, and 2 μg poly(dI:dC). The ^32^P-labeled oligonucleotide was added to the reaction mixture and incubated for 20 min at room temperature. Samples were electrophoresed on 6% acrylamide gels (made with 50 mM Tris buffer containing 380 mM glycine and 2 mM EDTA), followed by autoradiography.

For western blotting, the extracts were separated by 10% SDS-PAGE, followed by transfer to a nitrocellulose membrane (BioRad, CA, USA). After blocking with 5% fat-free milk protein in triethanolamine-buffered saline (TBS), pH 7.5, the membrane was incubated with the NF-κB-specific mouse anti-human mAb p65 (Santa Cruz Biotechnology, CA, USA). After washing, HRP-conjugated secondary antibody was added to the membrane in TBS containing 0.1% Tween 20. As an internal control, actin was used. An enhanced chemiluminescence detection system (ECL, Amersham Biosciences, Sweden) was used to visualize the protein bands.

### Statistical analysis

The mean and SD were calculated by Excel software (Microsoft). Student's t-test was used for statistical analysis, and *P *< 0.05 was considered significant.

## Abbreviations

MBL: mannan-binding lectin; APC: antigen presenting cell; CLR: collagen-like region; CRD: carbohydrate-recognition; domain; DC: dendritic cell; EMSA: electrophoretic mobility shift assay; HRP: horseradish peroxidase; HSA: human serum albumin; LPS: lipopolysaccharide; mAb: monoclonal antibody; MDC: monocyte-derived dendritic cell; pAb: polyclonal antibody; PRR: pattern recognition receptor; SP-A: surfactant proteins A; SP-D: surfactant proteins D; sTLR4: soluble form of recombinant extracellular domain of TLR4; TLR4: Toll-like receptor 4; TNF: tumor necrosis factor.

## Authors' contributions

**WMY **participated in the study design, carried out the cytokine measurements, contributed to the analysis of NF-κB activity, performed the statistical analyses for all studies and drafted the manuscript. **CY **participated in the study design and the analysis of cytokine expression. **ZYN **contributed to the analysis of DC maturation. **ZLY **prepared the MBL and contributed to the cultivation of cells. **LX **operated the flow cytometer in all the experiments and contributed to flow cytometric analysis. **CZL **designed the study, interpreted the data and drafted the manuscript. All authors read and approved the final manuscript.

## Supplementary Material

Additional file 1 **Fig. S1: MBL does not bind to smooth LPS**. Microtiter wells were coated with smooth LPS, rough LPS or mannan (10 μg/ml, 50 μl/well), and incubated with MBL at 37°C for 1 h. The binding of MBL was detected using anti-MBL mAb and HRP-labeled goat anti-mouse IgG as described under "Methods". The data shown are means ± S.E. of three experiments. Click here for file
