# Supplementary figures and images for: Mannan-binding lectin regulates dendritic cell maturation and cytokine production induced by lipopolysaccharide
Source: BMC Immunol. 2011 Jan 1;12:1. doi: 10.1186/1471-2172-12-1 (PMC3022617; doi:10.1186/1471-2172-12-1)

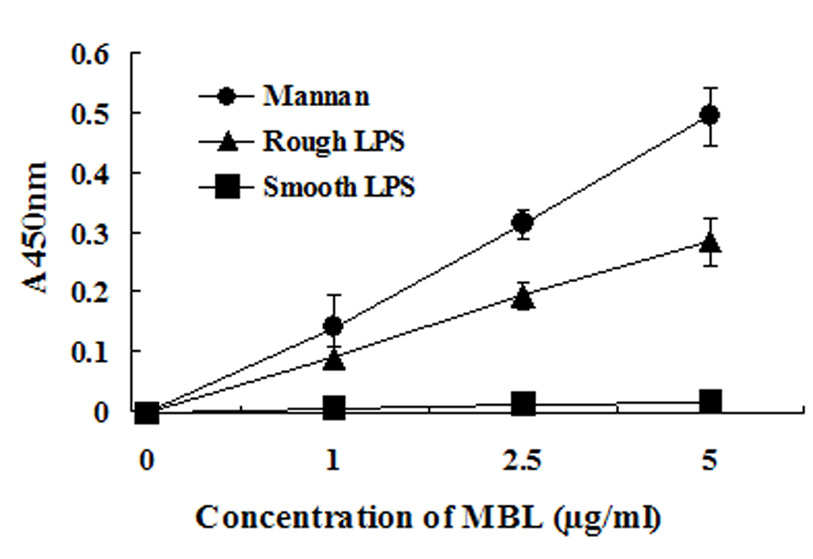

Supplement: Additional file 1 — Fig. S1: MBL does not bind to smooth LPS. Microtiter wells were coated with smooth LPS, rough LPS or mannan (10 μg/ml, 50 μl/well), and incubated with MBL at 37°C for 1 h. The binding of MBL was detected using anti-MBL mAb and HRP-labeled goat anti-mouse IgG as described under "Methods". The data shown are means ± S.E. of three experiments. [file 1471-2172-12-1-S1.TIFF]
